# Evaluation of the proficiency of trained non-laboratory health staffs and laboratory technicians using a rapid and simple HIV antibody test

**DOI:** 10.1186/1742-6405-2-5

**Published:** 2005-05-20

**Authors:** Koum Kanal, Thai Leang Chou, Ly Sovann, Yasuo Morikawa, Yumi Mukoyama, Kazuhiro Kakimoto

**Affiliations:** 1 National Maternal and Child Health Center, French street, Phnom Penh, Cambodia; 2 Japan International Cooperation Agency Maternal and Child Health Project in Cambodia, P.O. Box 613 Phnom Penh, Cambodia

## Abstract

In Cambodia, nearly half of pregnant women attend antenatal care (ANC), which is an entry point of services for prevention of mother-to-child transmission of HIV (PMTCT). However, most of ANC services are provided in health centres or fields, where laboratory services by technicians are not available. In this study, those voluntary confidential counselling and testing (VCCT) counsellors involved in PMTCT were trained by experienced laboratory technicians in our centre on HIV testing using Determine (Abbot Laboratories) HIV1/2 test kits through a half-day training course, which consisted of use of a pipette, how to process whole blood samples, and how to read test result. The trained counsellors were midwives working for ANC and delivery ward in our centre without any experience on laboratory works. The objective of this study was to assess the feasibility of the training by evaluating the proficiency of the trained non-laboratory staffs. The trained counsellors withdrew blood sample after pre-test counselling following ANC, and performed the rapid test. Laboratory technicians routinely did the same test and returned reports of the test results to counsellors. Reports by the counsellors and the laboratory technicians were compared, and discordant reports in two groups were re-tested with the same rapid test kit using the same blood sample. Cause of discordance was detected in discussion with both groups. Of 563 blood samples tested by six trained VCCT counsellors and three laboratory technicians, 11 samples (2.0%) were reported positive in each group, however four discordant reports (0.7%) between the groups were observed, in which two positive reports and two negative reports by the counsellors were negative and positive by the laboratory technicians, respectively. Further investigation confirmed that all the reports by the counsellors were correct, and that human error in writing reports in the laboratory was a cause of these discordant reports. These findings lead us the conclusion that the half-day training using the rapid and simple test was feasible for non-laboratory staffs to attain enough proficiency to implement VCCT services for PMTCT in resource-limited settings, and that human error was more likely to occur in laboratory before giving reports to counsellors.

## Findings

The National Health Statistics in Cambodia [1] estimated 89.4% of pregnant women in Cambodia to have given birth outside of health facilities, and approximately 48.4% of pregnant women to have at least once attended antenatal care (ANC) at health centres in 2003, which is an entry point of prevention of mother-to-child transmission of HIV (PMTCT) services. Furthermore, most of ANC services are provided in health centres or fields as one of outreach activities, where laboratory services by technicians are not available. In May 2003, the ministry of health in Cambodia started expanding PMTCT services following pilot projects in urban cities along with training of VCCT counsellors and laboratory technicians. Those pregnant women in ANC wishing to receive PMTCT services are offered voluntary confidential counselling and testing (VCCT) consisting of pre-test counselling, HIV testing and post-test counselling. However, some blood samples withdrawn by VCCT counsellors or some pregnant women that accepted VCCT need to be transported to a nearest laboratory by some means. For this reasons, some VCCT counsellors could be expected to provide HIV testing as well as pre-test and post-test counselling services to expand PMTCT services to cites where laboratory services are not available, and to increase uptake of pre-test and post-test counselling [2] if the HIV testing was accurately performed by VCCT counsellors in health centres or fields. In addition, rapid HIV testing is useful to improve access to learn HIV status in populations at high risk of HIV infection [3]. The aim of this study is to assess our training on HIV testing by comparing HIV testing performances of trained non-laboratory health staffs and laboratory technicians using a rapid and simple test.

Those VCCT counsellors involved in PMTCT in our centre were trained on HIV testing for half day using Determine (Abbot Laboratories) HIV1/2 test kits by experienced laboratory technicians. All the trained VCCT counsellors were midwives working for ANC and delivery ward in our centre, that hadn't have experience and knowledge on laboratory works. The contents of the training were how to use a pipette (30 minutes), how to process whole blood samples with chase buffer (90 minutes) including practical training, and how to read test result (60 minutes) according to the instructions provided by the manufacturer. The VCCT counsellors trained on the test withdrew blood sample with EDTA tubes from the clients' vein after pre-test counselling following ANC if informed consent to participate in PMTCT services was obtained, and performed the rapid test with whole blood and chase buffer. The rest of the blood sample was sent to a laboratory in our centre and centrifuged. The plasma samples were stored at 4 degrees centigrade and tested by the laboratory technicians with the same test kit on the next day. The test results by laboratory technicians were reported to counsellors in individual envelopes to keep confidentiality according to their routine. The rest of the plasma was stored in a freezer at -20 degrees centigrade for further examination. Only code numbers were used for the identification of samples with confidentiality, and printed on stickers in advance, which were used to label tubes and reports to minimize human error such as miswriting. Two reports from counsellors and laboratory technicians were compared, and discordant reports in two groups were re-tested with the same test kit in front of two groups using the same blood sample kept in the freezer, and cause of the discordance was detected in discussion with both groups.

Of 563 blood samples tested by six VCCT counsellors and three laboratory technicians, 11 samples (2.0%) were reported positive in each group, however four discordant reports (0.7%) between the groups were observed, in which two positive reports and two negative reports by the counsellors were negative and positive by the laboratory technicians, respectively (table 1). Further examination using the same test kit and blood samples of the discordant reports confirmed that two positive reports and two negative reports by the counsellors were positive and negative, respectively. In discussion with the groups, human error in writing reports in the laboratory was identified as a cause of these discordant reports.

**Table 1:** Comparison of reports of HIV test results between by VCCT counsellors and laboratory technicians. Further examination using the same test kit and blood samples confirmed that all the reports by the VCCT counsellors were correct, and that four discordant reports* were caused by human errors in the laboratory.

	HIV positive reports by VCCT counsellors	HIV negative reports by VCCT counsellors	Total
HIV positive reports by laboratory technicians	9	2*	11
HIV negative reports by laboratory technicians	2*	550	552
Total	11	552	563

In our study, the accuracy of reports by the VCCT counsellors scored 100%, which was higher than laboratory technicians (99.3%) though the Determine rapid and simple test requires one more step to use chase buffer for testing whole blood samples. This result showed that the half-day training for VCCT counsellors was feasible enough to provide satisfactory proficiency for non-laboratory health staffs using the rapid and simple test in order to implement the PMTCT services where laboratory technicians were not available. A study from the United States used the OraQuick rapid test to evaluate how well untrained persons with no laboratory experience can perform the HIV test, and concluded that they could achieve a level of satisfactory proficiency however they could not reach 100% accuracy [4].

The guidelines by World Health Organisation and National Centre for HIV/AIDS, Dermatology and STIs in Cambodia recommend either parallel testing or serial testing using two different test kits for VCCT activities [5,6]. The reason why our study, however, used only one test kit was that this study was the first step to assess our training and to evaluate accuracy of testing by the trained VCCT counsellors based on routine reports of HIV test results. Further study using two rapid test kits would be referred to follow the guidelines for the implementation of HIV testing by counsellors in Cambodia. Ziyambi, Z. et al. [7] reported that the combined sensitivity and specificity with two rapid tests by trained non-laboratory staff was 100% in Zimbabwe. However, our study was more practical by comparing two routine reports from laboratory technicians and the trained VCCT counsellors.

These findings suggest that the rapid and simple testing by non-laboratory health staffs trained through the half-day training could be recommended to expand PMTCT services providing same-day results. VCCT services for PMTCT with same-day results could expect to increase the access to HIV prevention and care [8].

Furthermore, it was realized in our study that human error was more likely to occur in process of the laboratory, even though the laboratory technicians were more skilled and experienced than VCCT counsellors and made effort to reduce the error by using the stickers. This happened not because of their capasity to process samples and to read the results, but because laboratory technicians have more complex recording and reporting processes before giving reports to counsellors. HIV testing by VCCT counsellors could reduce this risk as well. Whoever performs the test, however, importance of having strict procedures for quality assurance in testing cannot be overstated [9], and linkages to high-quality reference laboratory facilities for confirmatory testing and supervision system need to be carefully considered to expand PMTCT services using rapid tests [10].

In conclusion, the half-day training was sufficient enough for non-laboratory health staffs to attain proficiency of HIV testing and its report with a rapid and simple test kit, and it could contribute to enhance and to expand PMTCT activities more efficiently where laboratory technicians are not available. Further study using two kinds of test kits is needed to evaluate the feasibility of the training more practically.

## Competing interests

The author(s) declare that they have no competing interests.

## Authors' contributions

K. Kanal and K Kakimoto carried out data analysis and drafted this manuscript.

T. L. C. and Y. Mukoyama contributed to acquisition of data, and participated in coordination of study design to involve VCCT counsellors for PMTCT in this study.

L. S. and Y. Morikawa participated in this study to organise the training on HIV testing for the VCCT counsellors, and contributed to acquisition of data from the laboratory.

K. Kakimoto conceived of the design of this study with intellectual contribution.
